# Infiltrative and inflammatory systemic disorders involving the pituitary gland

**DOI:** 10.1007/s11102-026-01758-7

**Published:** 2026-09-16

**Authors:** Betina Biagetti, Pedro Marques, Silvana Sarria-Estrada, Manel Puig-Domingo, Marta Araujo-Castro, Mónica Marazuela

**Affiliations:** 1 Endocrinology & Nutrition Department, Hospital Universitario Vall d’Hebrón and Vall d’Hebron Research Institute (VHIR) ISS IR-HUVH-VHIR, Department of Medicine, Autonomous University of Barcelona, Reference Networks (Endo-ERN), Barcelona, 08035 Spain; 2 Pituitary Tumor Unit, Endocrinology Department, Hospital CUF Descobertas, Lisbon, Portugal; 3 Faculdade de Medicina, Universidade Católica Portuguesa, Lisbon, Portugal; 4 Neuroradiology Section, Radiology Department, Vall d’Hebron University Hospital, Vall d’Hebron Barcelona Hospital Campus, Universitat Autònoma de Barcelona, Barcelona, Spain; 5 Germans Trias Research Institute, Badalona, Spain; 6 Department of Endocrinology and Nutrition, Hospital Universitario Ramón y Cajal & Instituto de Investigación Ramón y Cajal (IRYCIS), Madrid, Spain; 7 Endocrinology & Nutrition Department, Hospital Universitario La Princesa, Instituto de Investigación Sanitaria Princesa &Universidad Autónoma de Madrid, Madrid, Spain

**Keywords:** Hypophysitis, Infiltrative pituitary disorders, Hypopituitarism, Pituitary stalk thickening, Granulomatous disease, Arginine vasopressin deficiency

## Abstract

The hypothalamic-pituitary region can be affected by a wide and heterogeneous group of infiltrative and inflammatory disorders, including granulomatous, histiocytic, storage, infectious, non-pituitary neoplastic, and autoimmune or inflammatory conditions collectively grouped as hypophysitis. Although individually rare, these disorders are increasingly recognized due to growing awareness, wider use of pituitary imaging, and the emergence of immune checkpoint inhibitor-induced hypophysitis. They typically present with hypopituitarism (often including arginine vasopressin deficiency, which, particularly when accompanied by pituitary stalk thickening, is highly suggestive of a non-adenomatous lesion) together with mass effect symptoms and, in many cases, systemic manifestations reflecting extrapituitary organ involvement. Neuroimaging findings, while rarely pathognomonic, can narrow the differential diagnosis, particularly when pituitary stalk involvement is present: symmetric stalk/gland enlargement with homogeneous enhancement suggests lymphocytic, IgG4-related, or granulomatous hypophysitis, whereas rim enhancement, necrosis, or bone-destructive lesions point toward infectious or neoplastic causes. A structured diagnostic work-up, combining clinical history, hormonal evaluation, targeted laboratory testing, pituitary and systemic imaging, and, when necessary, histopathological confirmation, is essential to establish an accurate diagnosis before committing patients to long-term immunosuppression or unnecessary pituitary surgery. Management requires two parallel strategies: prompt hormone replacement therapy, with urgent attention to corticotroph deficiency, and disease-specific treatment tailored to the underlying etiology, ranging from glucocorticoids and immunosuppressants to antimicrobial therapy, targeted oncologic agents, or surgery. Because pituitary deficits are frequently irreversible and several of these disorders follow a relapsing course, long-term multidisciplinary follow-up is warranted. This review summarizes the pathophysiology, clinical presentation, diagnostic approach and neuroimaging features, for infiltrative and inflammatory hypothalamic-pituitary disorders, aiming to facilitate their timely recognition and appropriate management.

## Introduction

Infiltrative and inflammatory pituitary disorders represent an important and often underrecognized category of non-adenomatous sellar pathology that can cause significant hypothalamic-pituitary dysfunction [1, 2]. They comprise a heterogeneous spectrum of diseases driven by autoimmune or immune-mediated inflammation, clonal cell proliferation, neoplastic infiltration, storage material deposition, or infectious organisms, all of which target or infiltrate the hypothalamic-pituitary region resulting in pituitary enlargement and varying degrees of anterior and posterior pituitary dysfunction. These disorders are important differential diagnoses for sellar masses because they frequently mimic pituitary adenomas on neuroimaging. Clinically, arginine vasopressin deficiency (AVP-D; formerly central diabetes insipidus) is often the earliest and most characteristic endocrine manifestation, commonly accompanied by anterior pituitary hormone deficiencies [2]. In contrast to pituitary adenomas, the combination of AVP-D and pituitary stalk thickening on magnetic resonance imaging (MRI) should strongly raise suspicion for an infiltrative or inflammatory process [2–6]. Although individually uncommon, this spectrum of infiltrative and inflammatory disorders encompasses a broad spectrum of systemic and primary pituitary diseases with markedly different prognoses and therapeutic approaches [7]. Early recognition is therefore essential to avoid diagnostic delay and initiate disease-specific treatment.

In this narrative review, we summarize the current evidence on infiltrative and inflammatory disorders affecting the hypothalamic-pituitary region, focusing on their pathophysiology, clinical presentation, diagnostic approach, neuroimaging features, and current therapeutic strategies to facilitate timely recognition and appropriate management, as well as for avoiding unnecessary pituitary surgery.

## Overview and classification

The 2024 Lancet classification of hypopituitarism distinguishes inflammatory causes (lymphocytic, granulomatous, IgG4-related, and immunotherapy-induced hypophysitis) from infiltrative causes (hemochromatosis, sarcoidosis, amyloidosis, histiocytosis), though considerable clinical overlap exists [2] (Table 1). Pituitary stalk thickening (PST) is a common neuroimaging thread linking many of these conditions [6, 8, 9]. In a large series of 321 patients with PST, AVP-D was the initial manifestation in 68.8%, and at least one anterior pituitary hormone deficit was found in 57.6%; hypothalamic involvement conferred a 7.3-fold increased odds of panhypopituitarism [10].

**Table 1:** Summary of inflammatory and infiltrative disorders of the pituitary gland per disorder category

Category	Disorders
Inflammatory/immune-mediated disorders	Lymphocytic hypophysitis, primary granulomatous hypophysitis, xanthomatous hypophysitis, IgG4-related hypophysitis, necrotizing hypophysitis, iatrogenic hypophysitis (immune checkpoint inhibitors and other drugs), paraneoplastic hypophysitis
Granulomatous disorders	Sarcoidosis (neurosarcoidosis), granulomatosis with polyangiitis, tuberculosis, fungal granulomatous infections, Tolosa-Hunt syndrome
Histiocytic disorders	Langerhans cell histiocytosis, Erdheim-Chester disease, Rosai-Dorfman disease
Storage disorders	Hemochromatosis (iron deposition), amyloidosis
Infectious disorders	Neurosyphilis (*Treponema pallidum*), neuroborreliosis (*Borrelia*), tuberculosis, fungal infections, viral infections, bacterial infections and abscess
Non-pituitary neoplastic disorders	Metastases (breast, lung, renal, thyroid, gastrointestinal cancers and melanoma), hematological malignancies (lymphoma, plasmacytoma, multiple myeloma), germinoma, meningioma, chordoid glioma, hemangioblastoma, hemangiopericytoma, chordoma, chondrosarcoma, ecchordosis physaliphora, bone giant cell tumor, pituicytoma

### Pituitary inflammatory/immune-mediated disorders

#### Lymphocytic hypophysitis

Lymphocytic hypophysitis (LH) is the most common histologic variant of primary hypophysitis [11] (Table 2). It is considered a rare inflammatory disorder characterized by autoimmune-mediated infiltration of adenohypophysis, neurohypophysis, or both [1]. Although traditionally considered a disease predominantly affecting women during late pregnancy or the postpartum period, it is now recognized across a broader spectrum of patients, including men and children [11–13]. In a systematic review of primary LH with 591 patients included, the mean age at presentation was 40.3 ± 6.2 years, with a wide range of age at presentation (3–81 years), and the study found a female predominance of 2.6:1 [14]. The condition may occur as an isolated entity or in association with other autoimmune diseases, such as autoimmune thyroiditis, type 1 diabetes mellitus, and autoimmune adrenal insufficiency [15]. In this regard, a history of autoimmune disease is commonly described in patients with LH (15–40% of patients) [12].

The clinical presentation of LH is highly variable and depends on the extent and location of pituitary involvement. Patients commonly present with headaches, visual disturbances due to compression of the optic chiasm, and varying degrees of anterior pituitary hormone deficiencies.

**Table 2:** Histopathological subtypes of primary hypophysitis

Type of primary hypophysitis	Epidemiology	Main histopathological features	Clinical characteristics	MRI findings	Treatment
Lymphocytic hypophysitis	Most common subtype of primary hypophysitis; predominantly affects women, especially during late pregnancy and the postpartum period; frequently associated with autoimmune diseases	Dense lymphocytic infiltration with plasma cells and fibrosis affecting the anterior pituitary, posterior pituitary, or stalk	Headache, visual disturbances, anterior hypopituitarism, and AVP-D; ACTH deficiency is common	Symmetric pituitary enlargement, stalk thickening, homogeneous and/or peripheral enhancement, and loss of the posterior pituitary bright spot	Hormone replacement; glucocorticoids for mass effect or severe symptoms; surgery in selected cases
Granulomatous hypophysitis	Rare; female predominance has been reported in idiopathic cases; may occur at any age	Noncaseating granulomas with multinucleated giant cells, histiocytes, lymphocytes, and plasma cells	More severe endocrine and non-endocrine manifestations than lymphocytic hypophysitis; headache, visual symptoms, hypopituitarism, and AVP-D are common	Pituitary enlargement and stalk thickening with homogeneous enhancement	Hormone replacement, glucocorticoids, and surgery for diagnosis or compressive symptoms
Xanthomatous hypophysitis	Exceptionally rare; predominantly affects young and middle-aged women	Lipid-laden foamy histiocytes, lymphocytes, plasma cells, and fibrosis	Headache and anterior pituitary dysfunction predominate; AVP-D is less common; possible association with Rathke’s cleft cysts	Frequently presents as a cystic or mixed cystic-solid sellar lesion	Surgical resection is often required; hormone replacement; glucocorticoids in selected cases
IgG4-related hypophysitis	Rare; predominantly affects middle-aged and older men; often associated with systemic IgG4-related disease	Dense lymphoplasmacytic infiltrate rich in IgG4-positive plasma cells with storiform fibrosis	Hypopituitarism, headache, visual symptoms, and AVP-D; frequently associated with pancreatic, salivary, retroperitoneal, or renal involvement	Diffuse pituitary enlargement, stalk thickening, homogeneous enhancement, and loss of the posterior bright spot	Glucocorticoids are first-line therapy; immunosuppressants or rituximab in refractory cases; hormone replacement
Necrotizing hypophysitis	Extremely rare; only isolated cases reported	Extensive pituitary necrosis associated with inflammatory infiltrates	Acute onset of severe headache, anterior hypopituitarism or panhypopituitarism, and occasional AVP-D	Enlarged pituitary gland and infundibulum with characteristic loss of central contrast enhancement	Hormone replacement; glucocorticoids in selected patients; surgery for diagnostic uncertainty or mass effect

**Abbreviations**: *ACTH* adrenocorticotropic hormone, *MRI* magnetic resonance imaging, *IgG4* immunoglobulin G4, *AVP-D* arginine vasopressin deficiency

**Fig. 1:**
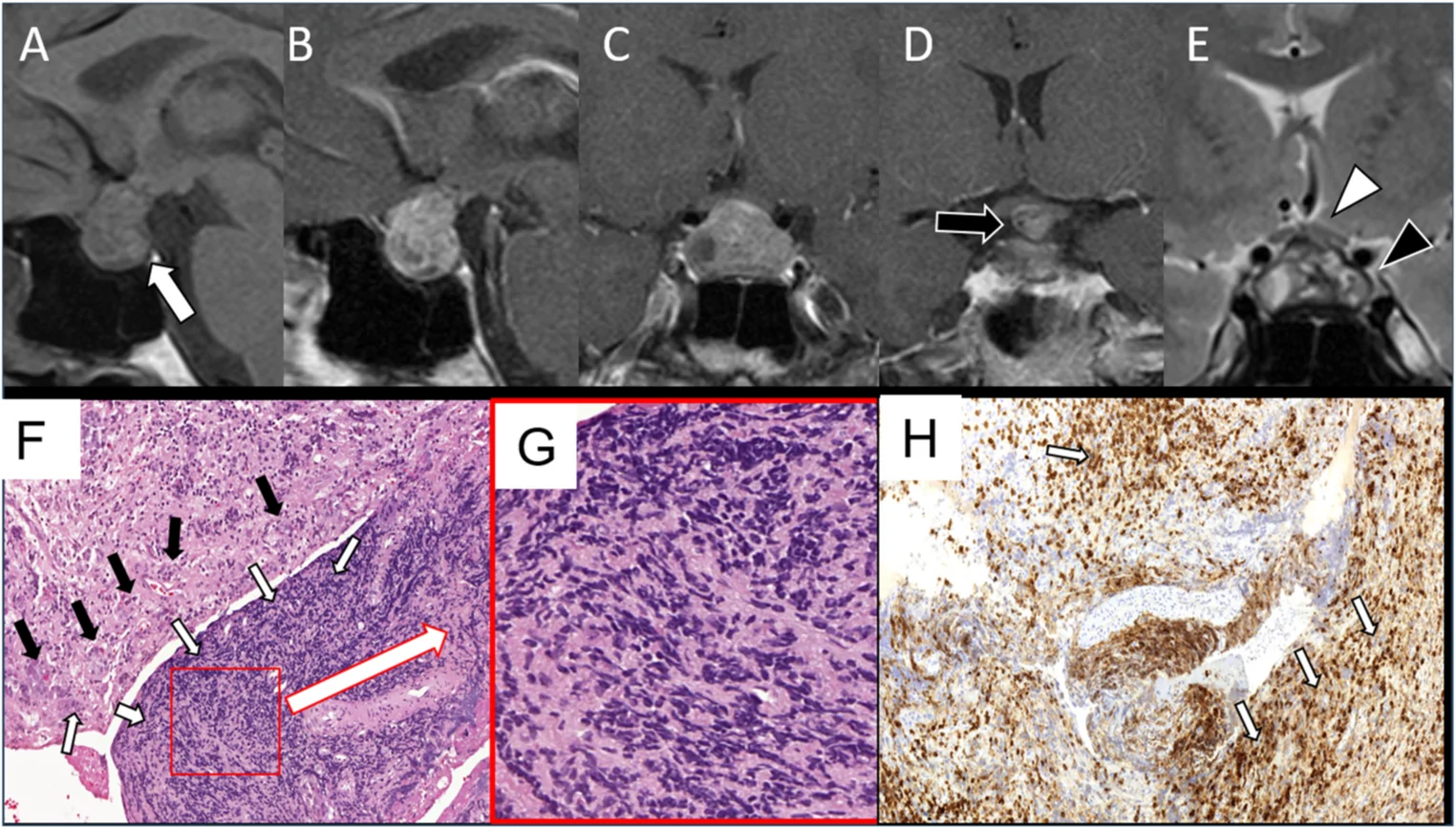
Lymphocytic hypophysitis. A 48-year-old woman with ACTH and TSH deficiency and suspected pituitary apoplexy. Absent T1WI bright spot (A, white arrow) with normal sella. Enlarged, heterogeneous pituitary gland (**A**–**C**, **E**) with thickened stalk (D, black arrow). Optic chiasm mass effect (D, white arrowhead) and peripheral T2 hyposignal (E, black arrowhead). Histopathology hematoxylin-eosin (**F**–**G**) and CD45 (**H**): pituitary glands (black arrows) and inflammatory infiltrate of mature lymphocytes (white arrows). Courtesy of Dra Elena Martinez, Neuropathology Unit Hospital Vall d’Hebron

It has been described that patients with pregnancy-related hypophysitis more frequently presented with chiasmal syndrome (50% of patients), particularly when onset occurred during pregnancy, and showed similar rates of headache and oculomotor nerve involvement [12]. ACTH deficiency is particularly frequent (70%) and may be life-threatening if unrecognized, followed by thyrotroph and gonadotroph deficiencies (approximately 60%) [1]. Involvement of the posterior pituitary and pituitary stalk may result in AVP-D, a feature that can help distinguish hypophysitis from pituitary adenomas. MRI typically reveals symmetric enlargement of the pituitary gland, PST, and homogeneous contrast enhancement frequently with peripheral predominance, although radiological findings may overlap with those of neoplastic and infiltrative disorders (Fig. 1).

The diagnosis of LH is based on a combination of clinical, hormonal, and imaging findings, while histopathological confirmation is reserved for selected cases with high degree of diagnosis uncertainty [16]. Recently, machine-learning analyses of texture features in pituitary MRI images have been explored as an additional tool to distinguish hypophysitis from pituitary adenomas [17]. Regarding treatment, management strategies depend on symptom severity and the presence of mass effect or hormonal dysfunction. High-dose glucocorticoids may induce rapid improvement in headache, visual symptoms, and pituitary enlargement, although recovery of endocrine function is often incomplete. Thus, a positive response to glucocorticoids may support the diagnosis of hypophysitis [18]. Steroid-sparing options such as azathioprine [18, 19] or the chimeric monoclonal antibody rituximab [19] have usually been reserved for recurrent disease. Long-term follow-up is essential because many patients require lifelong hormone replacement therapy and serial imaging to monitor disease progression or recurrence [16].

#### Primary granulomatous hypophysitis

**Fig. 2:**
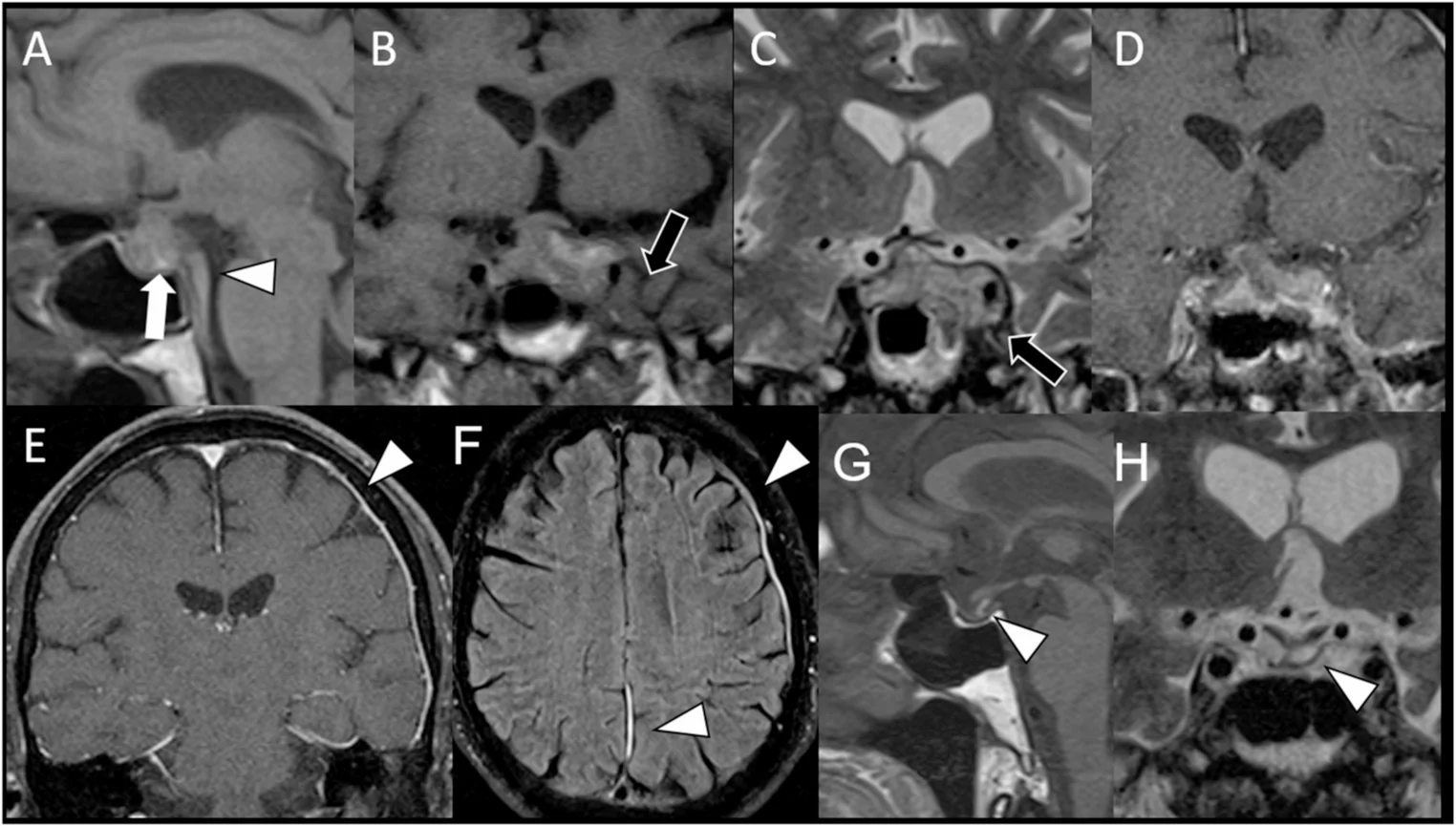
Granulomatous hypophysitis. A 69-year-old patient with subacute left ophthalmoparesis. Imaging shows an absent T1WI bright spot (**A**, white arrow) with a normal sella. A heterogeneous intrasellar-suprasellar mass with patchy internal hyperintensity suggests intralesional hemorrhage. The mass invades the cavernous sinuses (**B**-**C**, black arrow). There is heterogeneous enhancement of the mass (**D**) and pachymeningeal involvement (**E**-**F**, arrowhead). Following treatment, there is a marked decrease in gland size and stalk thickening (G-H, arrow, black arrowhead)

The second most frequent subtype of primary hypophysitis is granulomatous hypophysitis, accounting for approximately 20% of all cases [20] (Table 2). Primary granulomatous hypophysitis (PGH) is a rare inflammatory disease of the pituitary gland characterized by granulomatous destruction of pituitary tissue in the absence of an identifiable local or systemic cause [20]. A female predominance has been reported in a systematic review of idiopathic cases [20, 21] (Fig. 2).

Histologically, PGH is characterized by non-caseating granulomas composed of epithelioid histiocytes, multinucleated giant cells, lymphocytes, plasma cells, and variable fibrosis. Although the precise pathogenesis remains unknown, an autoimmune mechanism has been proposed based on its association with other autoimmune disorders and its response to immunosuppressive therapy [21]. Before establishing the diagnosis of PGH, secondary causes of pituitary granulomatous inflammation—including sarcoidosis, tuberculosis, granulomatosis with polyangiitis, syphilis, fungal infections, and Langerhans cell histiocytosis—must be carefully excluded through clinical, laboratory, microbiological, and radiological evaluation [21].

The clinical presentation is heterogeneous and reflects both local mass effects and pituitary hormone deficiencies. There are no distinguishing symptoms on presentation or radiologic feature to reliably differentiate granulomatous and lymphocytic hypophysitis [16]. Nevertheless, patients with PGH tend to present with more severe endocrine and extra-endocrine manifestations [13].

The diagnosis of PGH requires integration of clinical, endocrine, radiological, and pathological findings. Although advances in imaging such as diffuse enhancement of the infrasellar basisphenoid bone marrow [22] and clinical recognition have improved preoperative suspicion, definitive diagnosis frequently relies on histopathological examination of pituitary tissue demonstrating non-caseating granulomatous inflammation after exclusion of infectious and systemic granulomatous diseases [21]. The diagnosis of PGH is made following exclusion of secondary causes [21].

Treatment is individualized according to disease severity and diagnostic certainty. High-dose glucocorticoids constitute the mainstay of medical therapy and may reduce pituitary enlargement, improve headaches, and preserve visual function, although recovery of pituitary hormonal function is often incomplete. However, glucocorticoid therapy appears to be less effective than in LH while surgical resection leads to better symptom resolution [20]. Surgical intervention remains indicated in patients with significant neuro-ophthalmological compromise, uncertainty regarding the diagnosis, or failure to respond to medical treatment [21]. Because permanent hypopituitarism is common and disease recurrence has been described, long-term endocrine follow-up and serial pituitary imaging are recommended.

#### Xanthomatous hypophysitis

Xanthomatous hypophysitis (XH) is considered to be the rarest of the histological types [23] (Table 2). It is characterized histologically by the accumulation of lipid-laden foamy histiocytes, accompanied by variable degrees of lymphocytic infiltration, plasma cells, and fibrosis within the pituitary gland [24]. Since its first description in 1998, fewer than one hundred cases have been reported, making its epidemiology, pathogenesis, and natural history incompletely understood. XH predominantly affects young and middle-aged women and typically presents as an isolated inflammatory lesion of the sellar region [25]. Although its etiology remains uncertain, several hypotheses have been proposed, including autoimmune mechanisms and inflammatory responses triggered by leakage from ruptured Rathke’s cleft cysts, with some authors suggesting that XH may represent a secondary reaction rather than a distinct primary inflammatory disorder [20].

The clinical manifestations of XH depend on the extent of pituitary involvement and frequently overlap with those of other sellar lesions. The clinical symptoms appear to be milder and present for a longer duration in the patients compared to other types of hypophysitis [20]. Similarly, AVP-D is rarely reported with xanthomatous presentation [20]. MRI typically demonstrates a cystic or mixed cystic-solid sellar mass with variable contrast enhancement, often making preoperative differentiation from pituitary adenomas, Rathke’s cleft cysts, and craniopharyngiomas challenging [23].

The diagnosis of XH ultimately relies on histopathological examination, which reveals abundant foamy macrophages positive for CD68 intermixed with inflammatory cells and the absence of granulomatous inflammation or the storiform fibrosis characteristic of IgG4-related disease [23, 24]. Surgical resection is frequently performed because of diagnostic uncertainty and mass effect, although glucocorticoid therapy has been used in selected cases [23]. Despite favorable radiological outcomes following surgery, endocrine deficits often persist, necessitating long-term hormone replacement and clinical follow-up.

#### IgG4-related hypophysitis

IgG4-related hypophysitis (IgG4-RH) is a rare manifestation of IgG4-related disease (IgG4-RD), a systemic fibroinflammatory disorder characterized by tumefactive lesions, dense lymphoplasmacytic infiltrates enriched in IgG4-positive plasma cells, storiform fibrosis, and, in many cases, elevated serum IgG4 concentrations [16] (Table 2). Although IgG4-RH is considered rare, a recent retrospective histopathological review of cases previously diagnosed as LH revealed that 41% (12/29) were subsequently reclassified as IgG4-related hypophysitis [26] .

IgG4-RH predominantly affects middle-aged and elderly men, in contrast to LH, which is more common in women. Two literature reviews have reported that the median age is 55 years (62 years in men versus 38 years in women) [27, 28]. Pituitary disease may occur as an isolated manifestation, but it is more frequently associated with involvement of other organs, including the pancreas, salivary glands, retroperitoneum, kidneys, lungs, and lymph nodes. Therefore, the identification of hypophysitis should prompt a systematic evaluation for multisystem disease [29, 30].

The clinical presentation of IgG4-RH is heterogeneous and reflects the extent of anterior pituitary, posterior pituitary, and pituitary stalk involvement. Headache is observed in nearly one-third of patients with IgG4-RH, while visual pathway abnormalities are reported in 16–20% of cases. Overall, 75–78% of patients exhibit at least one pituitary hormone deficiency, with AVP-D representing the most common endocrine manifestation, followed by gonadotroph insufficiency [27, 28]. MRI typically reveals diffuse pituitary enlargement, PST, homogeneous contrast enhancement, and, occasionally, loss of the normal posterior pituitary bright spot. However, these findings are nonspecific and overlap with those observed in other inflammatory, infiltrative, and neoplastic sellar disorders [1].

The diagnosis of IgG4-RH relies on the integration of clinical, radiological, serological, and histopathological findings. However, its diagnosis is confirmed by characteristic histopathologic findings at pituitary biopsy that shows dense lymphoplasmacytic infiltration with increased numbers of IgG4-positive plasma cells and varying degrees of fibrosis, although pituitary biopsy is not always required when characteristic extrapituitary manifestations are present [29]. Serum IgG4 concentrations may support the diagnosis but lack sufficient sensitivity and specificity to be used in isolation [31]. It is recommended that laboratories reporting IgG4 should confirm freedom from prozoning (hook) effect [32].

**Fig. 3:**
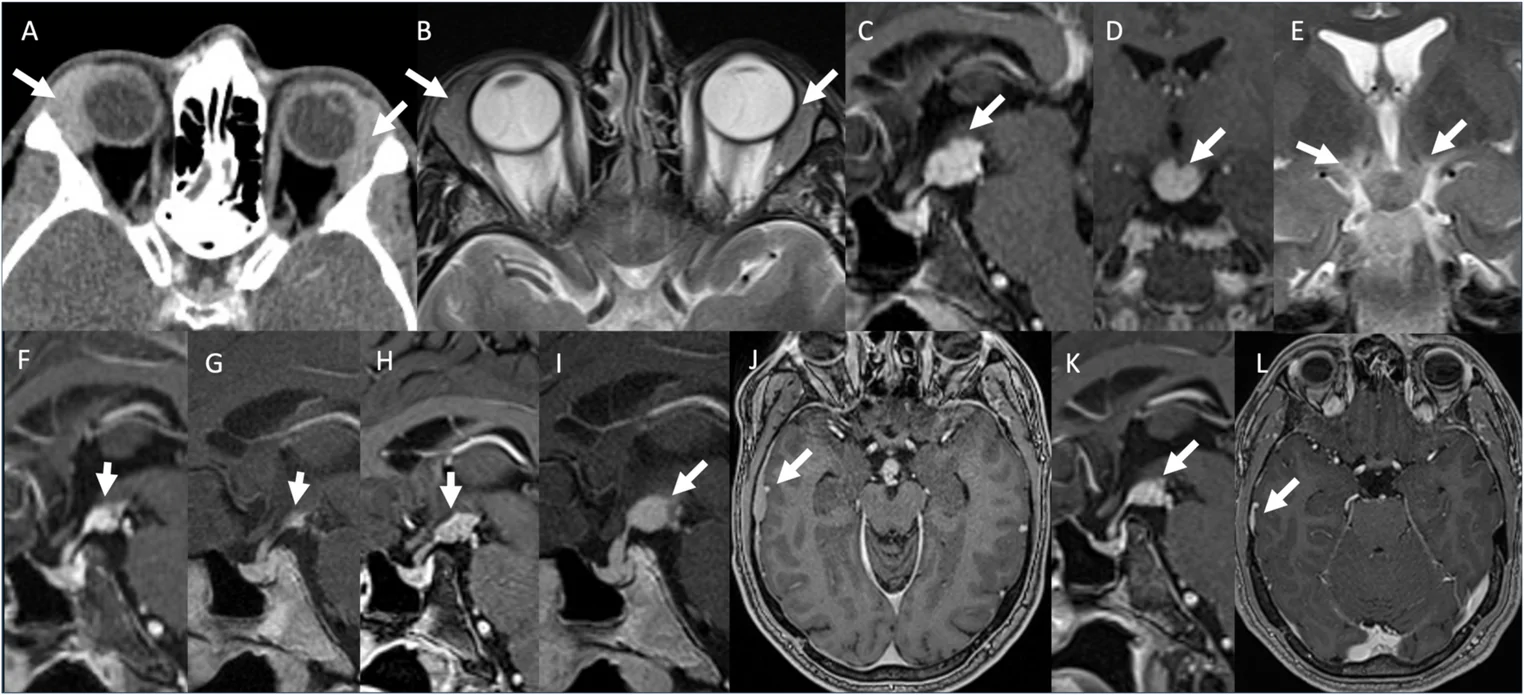
Radiological evolution of hypothalamic and dural involvement in a patient with IgG4-RD. A 33-year-old man with IgG4-RD initially presented with bilateral lacrimal gland enlargement, diagnosed as MALT lymphoma (**A**, orbital CT; **B**, orbital MRI; arrows). He subsequently developed hypothalamic involvement associated with hypogonadism (**C**, sagittal post-contrast T1-weighted image; **D**, coronal post-contrast T1-weighted image; **E**, coronal T2-weighted image showing a hypothalamic lesion with mild surrounding edema; arrows), which progressively decreased following treatment with rituximab and chlorambucil (**F**–**G**, arrows). At 3-year follow-up, the hypothalamic lesion had increased in size (**H**–**I**, arrows), with new right temporal dural thickening (**J**, arrow). An increase in serum IgG4 levels prompted reassessment of the lacrimal gland biopsy, which showed 80–100 IgG4-positive cells/HPF and an IgG4+/IgG+ ratio of 56–86%, fulfilling the 2019 ACR/EULAR classification criteria for IgG4-RD (24 points). Following treatment with an investigational monoclonal antibody, both the hypothalamic (**K**, arrow) and dural (**L**, arrow) lesions decreased in size. Abbreviations: CT, computed tomography; IgG4-RD, IgG4-related disease; MALT, mucosa-associated lymphoid tissue; MRI, magnetic resonance imaging; T1WI, T1-weighted image; T2WI, T2-weighted image

Glucocorticoids remain the first-line treatment and are generally associated with rapid improvement in pituitary enlargement and systemic manifestations, although recovery of pituitary function is often incomplete. In refractory or relapsing cases, steroid-sparing immunosuppressive agents and B-cell-depleting therapies, such as rituximab, may be considered, underscoring the importance of long-term multidisciplinary follow-up [33, 34]. Figure 3 illustrates the long-term radiological course of a patient with IgG4-RD, including the initial extrapituitary presentation, the response to B-cell-depleting therapy, and subsequent relapse with hypothalamic and dural involvement.

#### Necrotizing hypophysitis

Necrotizing hypophysitis (NH) is an exceptionally rare subtype of primary hypophysitis characterized histologically by extensive areas of ischemic necrosis accompanied by inflammatory infiltration of the pituitary gland. Since its initial description, only a limited number of cases have been reported in the literature, and consequently, its epidemiology, pathogenesis, and optimal management remain poorly understood [35, 36]. Although NH is generally considered a primary inflammatory disorder, its etiology has not been elucidated, and both autoimmune and vascular mechanisms have been proposed. Histopathological examination reveals varying degrees of necrosis associated with lymphocytic and plasma cell infiltration, in the absence of infectious, neoplastic, or systemic inflammatory diseases [37].

Patients typically present with acute-onset headache accompanied by anterior hypopituitarism or panhypopituitarism, frequently requiring long-term hormone replacement therapy. On magnetic resonance imaging, necrotizing hypophysitis usually manifests as diffuse enlargement of the pituitary gland and stalk, with a characteristic loss of central contrast enhancement [35]. The definitive diagnosis of NH generally requires pituitary biopsy or surgical resection [37]. Owing to the rarity of the condition, therapeutic strategies are largely based on case reports and small case series. Management includes hormone replacement therapy to correct pituitary deficiencies, glucocorticoids in selected patients to control inflammation, and neurosurgical intervention in cases of diagnostic uncertainty or progressive neuro-ophthalmological compromise [1].

#### Iatrogenic hypophysitis secondary to immune checkpoint inhibitors (ICIs) and other drugs

Monoclonal antibodies targeting cytotoxic T-lymphocyte-associated antigen 4 (CTLA-4), programmed cell death protein 1 (PD-1), and programmed death ligand 1 (PD-L1), including ipilimumab, nivolumab, pembrolizumab, and atezolizumab, have all been implicated in iatrogenic hypophysitis [38]. Hypophysitis occurs most frequently with anti-CTLA-4 agents and combination regimens, reflecting differences in the underlying immunological mechanisms. Experimental evidence suggests that CTLA-4 blockade may induce complement-mediated pituitary injury, resulting in lymphocytic infiltration and selective destruction of hormone-secreting cells [38]. The incidence of hypophysitis has been reported to reach up to 20% in patients receiving anti-CTLA-4 therapy, whereas it is considerably lower with anti-PD-1 and anti-PD-L1 agents, ranging from 0.2% to 6% [39, 40]. The risk increases substantially with combination regimens, with incidences of up to 30–40%. Furthermore, hypophysitis typically develops earlier during treatment with anti-CTLA-4 agents (approximately 2–3 months after treatment initiation) than with anti-PD-1 or anti-PD-L1 therapies, in which onset generally occurs after 4–6 months [41, 42].

The clinical presentation of iatrogenic hypophysitis is highly variable and depends on the causative agent and the extent of pituitary involvement. The pathogenesis of anti-CTLA-4-associated hypophysitis involves T-cell-mediated pituitary inflammation, which typically leads to combined deficiencies of the anterior pituitary axes, including corticotroph, thyrotroph, and gonadotroph dysfunction, as well as transient enlargement of the pituitary gland on MRI. Conversely, hypophysitis related to anti-PD-1 and anti-PD-L1 agents usually presents with isolated corticotroph deficiency, often in the absence of overt symptoms or characteristic imaging findings, underscoring the need for routine hormonal screening [43–45]. MRI may demonstrate diffuse pituitary enlargement, stalk thickening, and homogeneous contrast enhancement, although normal imaging findings are not uncommon, particularly in cases associated with PD-1 and PD-L1 inhibitors. In contrast to primary autoimmune hypophysitis, AVP-D is relatively uncommon in ICI-induced disease [45].

Although ICIs account for most contemporary cases, hypophysitis has also been described in association with other therapeutic agents. These include interferon-α, interleukin-2, anti-tumor necrosis factor agents, and, more rarely, medications such as tyrosine kinase inhibitors and immune-modulating therapies [1, 46]. Management consists primarily of hormone replacement therapy, particularly glucocorticoid supplementation in patients with adrenal insufficiency, whereas high-dose corticosteroids are generally reserved for severe cases with significant mass effect or neurological symptoms. Since endocrine recovery is often incomplete, long-term endocrine follow-up remains essential.

#### Paraneoplastic hypophysitis

The proposed mechanism underlying paraneoplastic hypophysitis involves aberrant expression of PIT-1 in neoplastic tissue, resulting in the activation of PIT-1-specific cytotoxic T cells and the subsequent generation of anti-PIT-1 antibodies [47]. Rather than anti-PIT-1 antibodies, PIT-1-reactive cytotoxic T cells appear to be responsible for the selective injury of GH-, prolactin-, and TSH-secreting pituitary cells. To date, paraneoplastic hypophysitis has been reported in only nine patients, including four cases associated with thymoma and five linked to a variety of other malignancies [48]. All patients had GH and prolactin deficiency, and very low TSH levels. All patients showed a normal or slightly atrophic pituitary gland on MRI [48].

### Infiltrative disorders

#### Sarcoidosis

**Fig. 4:**
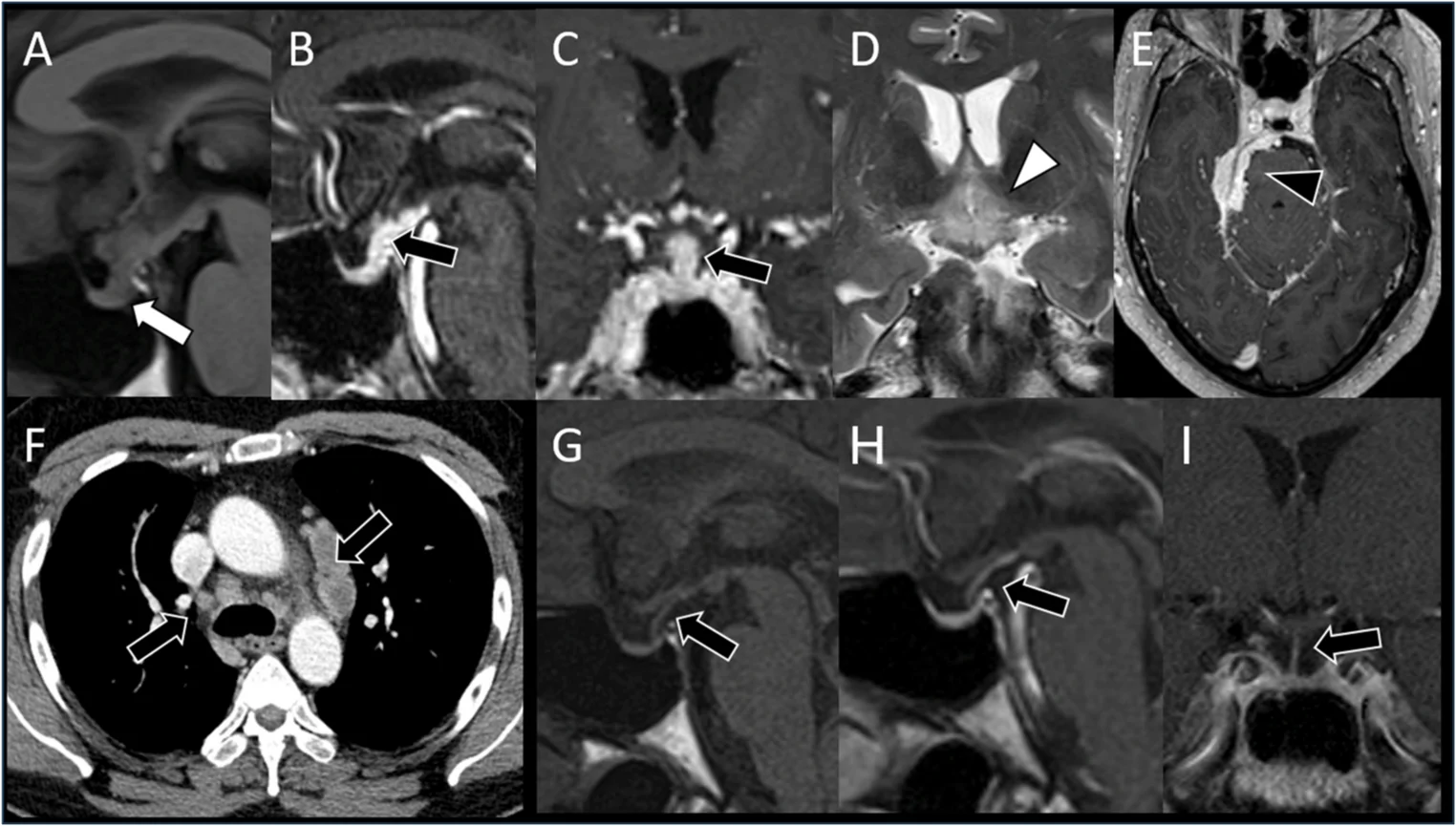
Sarcoidosis. A 45-year-old woman with headache and panhypopituitarism. Imaging shows a normal sella and absence of the posterior pituitary T1WI bright spot (**A**, white arrow). Contrast-enhanced images reveal a thickened stalk (**B**–**C**, black arrow) and pachyleptomeningeal enhancement (**E**, black arrowhead). Coronal T2WI demonstrates hypothalamus/optic chiasm edema (**D**, white arrowhead). Contrast-enhanced chest CT shows mediastinal lymphadenopathy (arrow). Two-year follow-up post-corticosteroid therapy shows normalization of the pituitary stalk (G–I, arrows)

Sarcoidosis is a multisystemic inflammatory disease of unknown etiology characterized by organ non-caseating granulomatous inflammation. The lungs are most affected (90%), followed by liver (20–30%), eye (10–30%), lymph nodes (10–20%), skin (15%), spleen (10%), central nervous system (CNS) (5–15%) and heart (2–5%) [1]. In a recent retrospective cohort, about one-sixth of patients with involvement of the CNS may have the hypothalamus, infundibulum or pituitary gland affected; however, only about 39% were asymptomatic [49]. Neurosarcoidosis diagnosis is challenging and often requires histopathological confirmation because serum angiotensin-converting enzyme and imaging lack sufficient sensitivity and specificity [50, 51]. Hypopituitarism is common in pituitary sarcoidosis and is the initial manifestation in more than half of affected patients. Central hypogonadism is the most common abnormality (75–90%), followed by secondary hypothyroidism (40–65%), growth hormone (GH) deficiency (50%) and secondary adrenal insufficiency (33–49%). AVP-D deficiency occurs in 25–50%, although polydipsia and polyuria without AVP-D may also occur due to the hypothalamic inflammation of the thirst center. Hyperprolactinemia has been described in 30–77% of patients [1, 50, 52]. On imaging, CNS sarcoid lesions typically demonstrate contrast enhancement on computed tomography (CT) without associated edema or calcifications, and MRI usually shows gadolinium-enhancing lesions with pituitary stalk thickening [50, 53, 54]. Additional inflammatory sarcoid lesions can be found in the CNS (especially in the cranial nerves and meninges), as well as in extracranial organs, which may support the diagnostic work-up [49]. Glucocorticoids and other immunosuppressants (including tumor necrosis factor (TNF) inhibitors) may reduce infiltrative lesions but rarely reverse established hypopituitarism. Refractory pituitary lesions may require transsphenoidal surgery, and anterior and posterior pituitary deficiencies should be managed with appropriate replacement therapy [49, 50, 52]. Figure 4 shows a patient with systemic sarcoidosis affecting the pituitary gland.

#### Granulomatosis with polyangiitis

Granulomatosis with polyangiitis (GPA) is a systemic small-vessel vasculitis characterized by pauci-immune necrotizing inflammation. Pituitary involvement occurs in approximately 1–2% of patients [55, 56]. Although more than 90% of patients with active disease are positive for anti-neutrophil cytoplasmic antibodies (ANCAs), histopathological confirmation may be necessary [55, 56]. Pituitary dysfunction is the initial manifestation in nearly half of patients with pituitary GPA. AVP-D occurs in up to 88% of patients, whereas anterior pituitary dysfunction is less frequent, including central hypogonadism (54%), secondary hypothyroidism (37%), secondary adrenal insufficiency (28%), GH deficiency (15%) and hyperprolactinemia (approximately one-third of patients) [56]. Pituitary MRI typically shows pituitary enlargement, a sellar mass with peripheral heterogeneous or homogeneous enhancement, areas of central necrosis, stalk thickening, cystic pituitary changes, and loss of the normal neurohypophysis hyperintensity [55–57]. Treatment with glucocorticoids combined with cyclophosphamide or rituximab is generally effective [56, 58, 59]. Pituitary deficiencies often persist despite adequate systemic responses and structural resolution of the lesions, AVP-D might reverse in two-thirds of cases, and visual outcomes are variable. Surgical decompression may be necessary to relieve compressive symptoms [55–57, 60] .

#### Tuberculosis

Tuberculosis can involve the hypothalamic-pituitary region and present as a sellar tuberculoma or tubercular hypophysitis masquerading as pituitary adenoma or pituitary apoplexy [61]. Diagnosis is particularly challenging in the absence of systemic tuberculosis, which occurs in about 40% of patients with tuberculous hypophysitis [62, 63]. Importantly, the absence of systemic manifestations or typical radiological findings does not exclude pituitary tuberculosis, as also highlighted in a recent systematic review of pediatric sellar/suprasellar tuberculosis [62] Diagnostic evaluation should include interferon-gamma release assays (IGRA), tuberculin skin test and/or bacteriological analysis for tuberculosis (e.g. sputum, tissue, lung or cerebrospinal fluid, as appropriate [1, 64]), as well as lung CT or ^18^F-fluorodeoxyglucose positron emission tomography (^18^F-FDG-PET) [65, 66]. In a study including 115 patients with tuberculous meningitis, pituitary dysfunction was identified in 54%, most commonly central hypogonadism (34%), followed by hyperprolactinemia (23%), secondary hypothyroidism (17%), secondary adrenal insufficiency (13%), and GH deficiency (8%); syndrome of inappropriate antidiuretic hormone secretion (SIADH) was reported in 10% of patients, whereas AVP-D was not reported [67]. Pituitary MRI typically shows a sellar/suprasellar mass with focal non-enhancing areas and absence of the posterior pituitary hyperintensity [63, 68, 69]. Anti-tuberculosis therapy is the mainstay of treatment but rarely restores pituitary function; surgery may be required in selected cases.

#### Tolosa-Hunt syndrome

Tolosa-Hunt syndrome is a rare cause of painful ophthalmoplegia resulting from idiopathic granulomatous inflammation of the cavernous sinus and/or superior orbital fissure. Patients typically present with retro-orbital pain, diplopia, ptosis and vision loss; anterior or posterior pituitary deficiencies may also occur. MRI characteristically demonstrates enlargement and contrast enhancement of the cavernous sinus extending through the superior orbital fissure to the orbital apex, although sellar inflammatory lesions may mimic a pituitary adenoma. The diagnosis is based on the characteristic clinical presentation and MRI findings, and histopathological confirmation is generally unnecessary. Glucocorticoids are the mainstay treatment, relieving the pain within 72 h, while ophthalmoplegia may resolve more slowly or persist [70–72] .

#### Histiocytic disorders

##### Langerhans cell histiocytosis

**Fig. 5:**
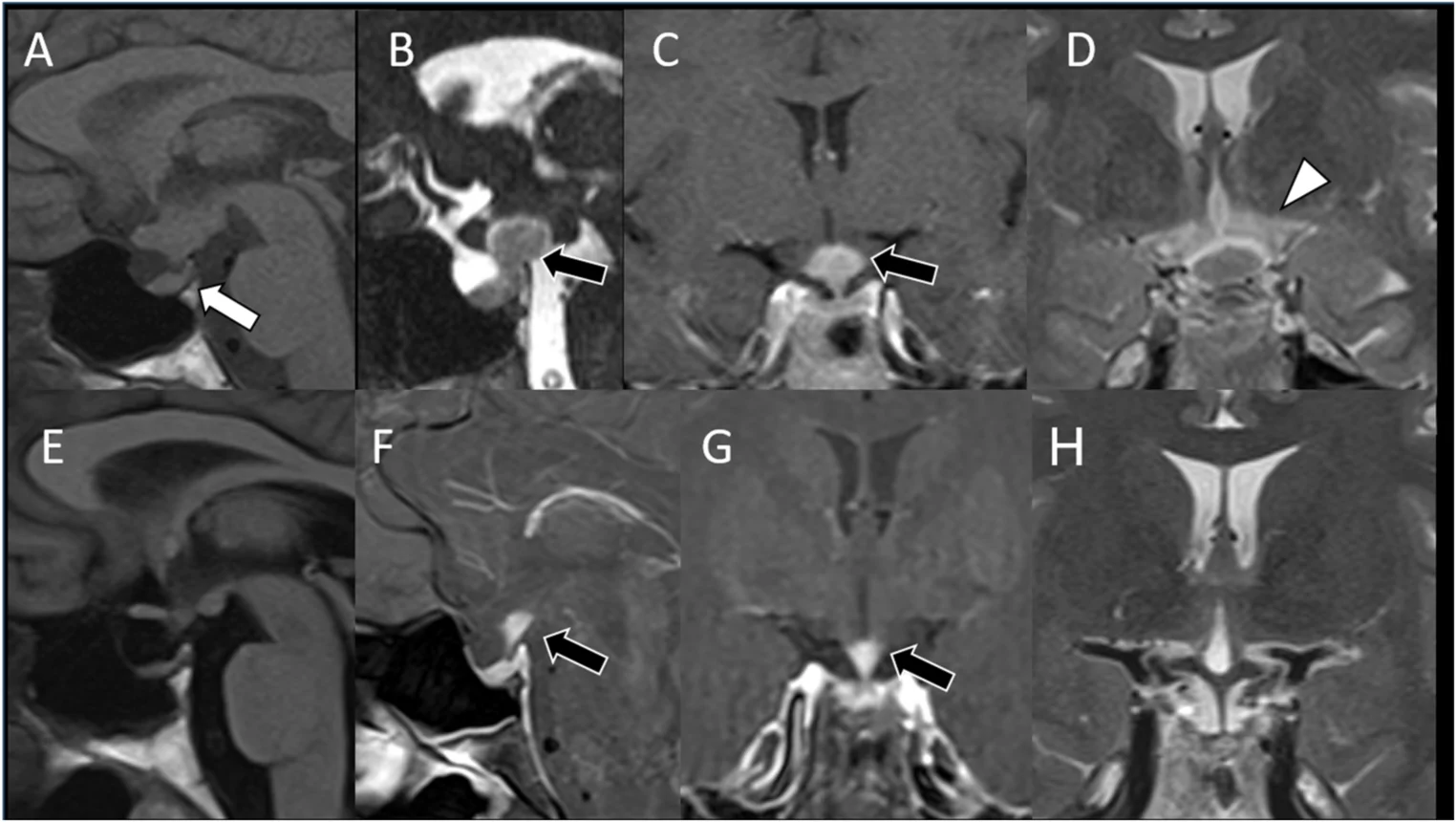
Langerhans cell histiocytosis. A 41-year-old woman with arginine vasopressin deficiency, hypogonadism, and hyperprolactinemia. Imaging shows a normal sella turcica with an absent posterior pituitary T1WI bright spot (**A**, white arrow), thickened stalk (**B**–**C**, black arrow), and hypothalamic involvement (D, arrowhead). Coronal T2WI shows hypothalamus/optic chiasm edema (**D**, white arrowhead). Two-year follow-up after treatment indicates decreased pituitary stalk thickening (**F**–**G**, arrows). Langerhans cell histiocytosis with BRAF V600E mutation

Langerhans cell histiocytosis (LCH) is a multisystemic disorder characterized by clonal histiocytic lesions typically affecting the lungs (40–50%), bones (30–50%), CNS (15–20%), liver (10–15%) and skin (5–10%), and also with frequent involvement of the hypothalamic-pituitary region [1]. LCH diagnosis is usually suspected when other multisystem features and/or AVP-D occur. Histopathological confirmation from the most accessible lesion is recommended when feasible. Mutations in *BRAFV600E* and MAPK-ERK pathway can be found in about half of cases [1, 73, 74]. AVP-D is the most common pituitary disturbance, occurring in 50–70% of patients [75–77]. Anterior deficiencies can occur in 20–63% of LCH patients, more often in childhood-onset cases, predominantly GH and gonadotrophin deficiencies. Hyperprolactinemia, due to hypothalamic disturbance or stalk compression, may contribute further to gonadotrophin suppression [75–77]. In a recent study encompassing 48 patients with hypothalamic-pituitary LCH, 69% of the patients had AVP-D, 42% had central hypogonadism, 25% had secondary hypothyroidism, and 12.5% secondary adrenal insufficiency [77]. CT and MRI typically show an enhancing hypothalamic-pituitary mass, often associated with pituitary stalk thickening and loss of the normal neurohypophysis hyperintensity in patients with AVP-D (Fig. 5). ^18^F-FDG-PET is useful for identifying active lesions and monitoring treatment response [75, 76, 78]. Treatment includes glucocorticoids, chemotherapy, radiotherapy, and, in selected cases, targeted therapy [25, 75]. Tyrosine kinase inhibitors, particularly vemurafenib, shows promising results in patients with *BRAF* V600E mutations and pathological MAPK activation, with response rates varying from 41 to 100%. Endocrine dysfunction rarely recovers after hypothalamic involvement in LCH patients [73, 78, 79].

##### Erdheim-Chester disease

Erdheim-Chester disease (ECD) is a rare multisystem non-Langerhans cell histiocytosis characterized by clonal myeloid proliferation with prominent inflammatory infiltrate [80]. Most clones are predominantly driven by *BRAF* and *NRAS*. *BRAFV600E* mutations have been reported in about 50% of ECD patients. Organs/sites frequently affected include bones (> 80%), kidney (60%), CNS (50%), lungs (< 50%), aorta (40%), heart (30%), retroperitoneum (30%) and skin (20%) [1, 80]. Typical manifestations include bilateral osteosclerotic lesions of the long bones, circumferential aortic wall thickening, and retroperitoneal fibrosis. Diagnosis requires histopathological confirmation demonstrating CD68+/CD1a-/S100-low, Langerin (CD207)-foamy or lipid-laden histiocytes [81–83]. Hypothalamic-pituitary involvement may result in partial hypopituitarism (91%), panhypopituitarism (18%), and AVP-D(33%). Pituitary imaging abnormalities can be found in one-third of cases, typically absent neurohypophysis hyperintensity or stalk thickening [80, 82, 84]. AVP-D may precede systemic manifestations by several years, whereas radiological abnormalities can develop months to years later, suggesting gradual progression from microscopic to clinically overt pituitary diseases [80, 82]. Treatment options include interferon, vinblastine and targeted therapies, including BRAF and MEK pathway inhibitors. Such treatments may reduce hypothalamic-pituitary lesions in ECD, but established pituitary dysfunction is often irreversible [73, 82, 85] .

##### Rosai-Dorfman disease

Rosai-Dorfman disease is a rare myeloproliferative disorder of histiocytes with foamy histiocytes and emperipolesis (presence and movement of one cell in the cytoplasm of another). Rosai-Dorfman disease typically presents with bilateral painless cervical lymphadenopathy, accompanied by fever, weight loss, and night sweats. CNS involvement accounts for about 5% of all cases; however, extranodal Rosai-Dorfman lesions (including in the pituitary) without lymphadenopathy are extremely rare. Common symptoms in patients with pituitary involvement include headache, visual impairment and pituitary dysfunction [86, 87]. On MRI, Rosai-Dorfman disease lesions are typically isointense on T1 and low to isointense in T2, and there is uniform enhancement [88] .

### Storage and infectious disorders

Storage disorders comprise a heterogeneous group of conditions in which metals like iron or protein aggregates accumulate within the pituitary gland, which may impair hormone secretion with or without a discrete mass lesion. Infectious causes are similarly rare and typically result from hematogenous dissemination or contiguous spread from adjacent structures, most often in immunocompromised or untreated patients, and can likewise mimic a pituitary adenoma or hypophysitis on imaging. Table 3 summarizes the storage entities (hemochromatosis [89, 90] and amyloidosis [91]) and the main infectious entities (syphilis [92], bacterial infections including abscess [93], fungal infections [51, 94], and viral infections [95–97]), together with their characteristic clinical, radiological and histopathological features and current management.

**Table 3:** Storage and infectious disorders: key distinguishing features

Entity	Typical clinical/radiological clues	Treatment
Hemochromatosis	Central hypogonadism from predominant gonadotroph involvement (subnormal testosterone with low/inappropriately normal gonadotrophins), although primary hypogonadism may also occur due to iron accumulation in the gonads; secondary adrenal insufficiency may occur, while somatotroph and thyrotroph axes are usually preserved; reduced T2-weighted pituitary signal ± increased gland volume on MRI; iron deposits in gonadotrophs (less commonly lactotrophs) on histopathology.	Iron depletion therapy (phlebotomy/chelation) plus hormone replacement; pituitary dysfunction often persists despite iron depletion
Amyloidosis	Usually vascular/interstitial amyloid deposition without a discrete pituitary mass; pituitary dysfunction is rare; more commonly associated with thyroid, adrenal, pancreatic or testicular involvement	Treatment of the underlying systemic amyloidosis; hormone replacement only if a deficiency is confirmed (rare)
Syphilis (neurosyphilis)	Sexually transmitted (Treponema pallidum); positive treponemal serology or tissue diagnosis; hypophysitis with pituitary dysfunction; hypothalamic/pituitary gumma may cause mass effect mimicking an adenoma	Antibiotics (penicillin-based regimens)
Bacterial infections / pituitary abscess	Hematogenous spread or contiguous extension (e.g., meningitis, sphenoid sinusitis), mostly Staphylococcus/Streptococcus; headache, visual impairment and pituitary dysfunction (anterior hypofunction ~ 82%, AVP-D ~ 48%); fever/leukocytosis/meningismus in about one-third; sellar mass with peripheral (rim) enhancement on MRI; diagnosis often intraoperative	Surgical drainage plus targeted antibiotics; hormone deficiencies often persist; mortality ~ 8%
Fungal infections	Aspergillus, Nocardia or Candida, mostly in immunocompromised patients; AVP-D ± anterior deficiencies (central hypogonadism common); sellar/parasellar mass with rim enhancement mimicking an adenoma; septate hyphae on Grocott’s stain	Antifungal therapy; surgery may be required
Viral infections	Herpes simplex, varicella, enterovirus (meningitis); influenza, Coxsackie (meningoencephalitis); cytomegalovirus (encephalitis); hypopituitarism may develop acutely or months later; hypophysitis with hypopituitarism rarely reported after COVID-19 infection or vaccination	Antiviral therapy where applicable; hormone replacement and supportive care as needed

### Non-pituitary neoplastic disorders

A wide range of non-pituitary neoplasms [98], metastatic [99–101], or arising from adjacent skull-base structures [102], can infiltrate or compress the hypothalamic-pituitary region and mimic a pituitary adenoma or hypophysitis. Although individually rare, they should be suspected whenever imaging shows atypical features [103] (asymmetric enlargement, bone destruction, a dural tail, or extension beyond the sella) or when the clinical course is rapid or uncommon. Table 4 summarizes the entities most frequently encountered, their characteristic clinical and radiological clues, and standard treatment. Unlike the infiltrative and inflammatory disorders discussed above, these lesions do not respond to immunosuppression; diagnosis usually requires histological confirmation, and management is entity-specific, combining surgery, radiotherapy and/or systemic oncological therapy as appropriate.

**Table 4:** Non-pituitary neoplastic disorders: key distinguishing features

Entity	Typical clinical/radiological clues	Treatment
Pituitary metastases	Known primary cancer (breast, lung most common); rapid-onset AVP-D and/or visual loss; posterior pituitary/stalk predilection	Surgery (diagnosis/debulking), radiotherapy, systemic therapy per primary condition
Lymphoma (primary or secondary)	Cranial nerve palsies, hyperprolactinemia; non-specific MRI mimicking adenoma or hypophysitis	Systemic chemotherapy ± radiotherapy
Plasmacytoma	Headache, diplopia; may be first manifestation of multiple myeloma	Surgery, radiotherapy, chemotherapy
Germinoma	Children/young adults, male predominance; AVP-D plus anterior deficits; pineal gland co-involvement; ↑hCG/α-fetoprotein	Radiotherapy ± chemotherapy (radiosensitive)
Meningioma	Female predominance; homogeneous enhancement with a dural tail sign	Surgery and/or radiotherapy if symptomatic
Chordoid glioma	Anterior third ventricle; T2-hyperintense, homogeneous enhancement	Partial resection plus adjuvant radiosurgery preferred (radical surgery carries high morbidity)
Hemangioblastoma	Younger patients; consider von Hippel–Lindau disease; cystic lesion with mural nodule	Surgery if symptomatic or progressive
Hemangiopericytoma	Aggressive; broad dural attachment, heterogeneous enhancement	Surgery plus adjuvant radiation
Chordoma	Clival origin; T2-hyperintense, bone destruction; physaliphorous cells on biopsy	Surgery plus postoperative radiotherapy (chemo-resistant)
Chondrosarcoma	Petroclival synchondrosis (lateral to clivus); can mimic chordoma	Surgery plus adjuvant radiotherapy
Ecchordosis physaliphora	Retroclival, usually incidental; pathognomonic bony stalk	Surgical resection only if symptomatic
Giant cell tumor of bone	Osteolytic lesion on CT; multinucleated giant cells on biopsy	Surgery, radiotherapy, bisphosphonates/denosumab
Pituicytoma	Neurohypophysis/infundibulum origin; TTF-1 positive	Gross total surgical resection

## Clinical approach: from suspicion to management

A pragmatic clinical approach is essential to differentiate inflammatory and infiltrative disorders of the hypothalamic-pituitary region from pituitary adenomas and to guide timely, disease-specific management. Clinical suspicion should be raised whenever AVP-D and/or PST accompany anterior pituitary hormone deficiencies, particularly when the pattern, onset, or associated systemic features (skin, lung, renal, ocular, or neurological involvement; lymphadenopathy; bone lesions; recent initiation of immunotherapy or other implicated drugs) diverge from the typical presentation of a pituitary adenoma.

**Fig. 6:**
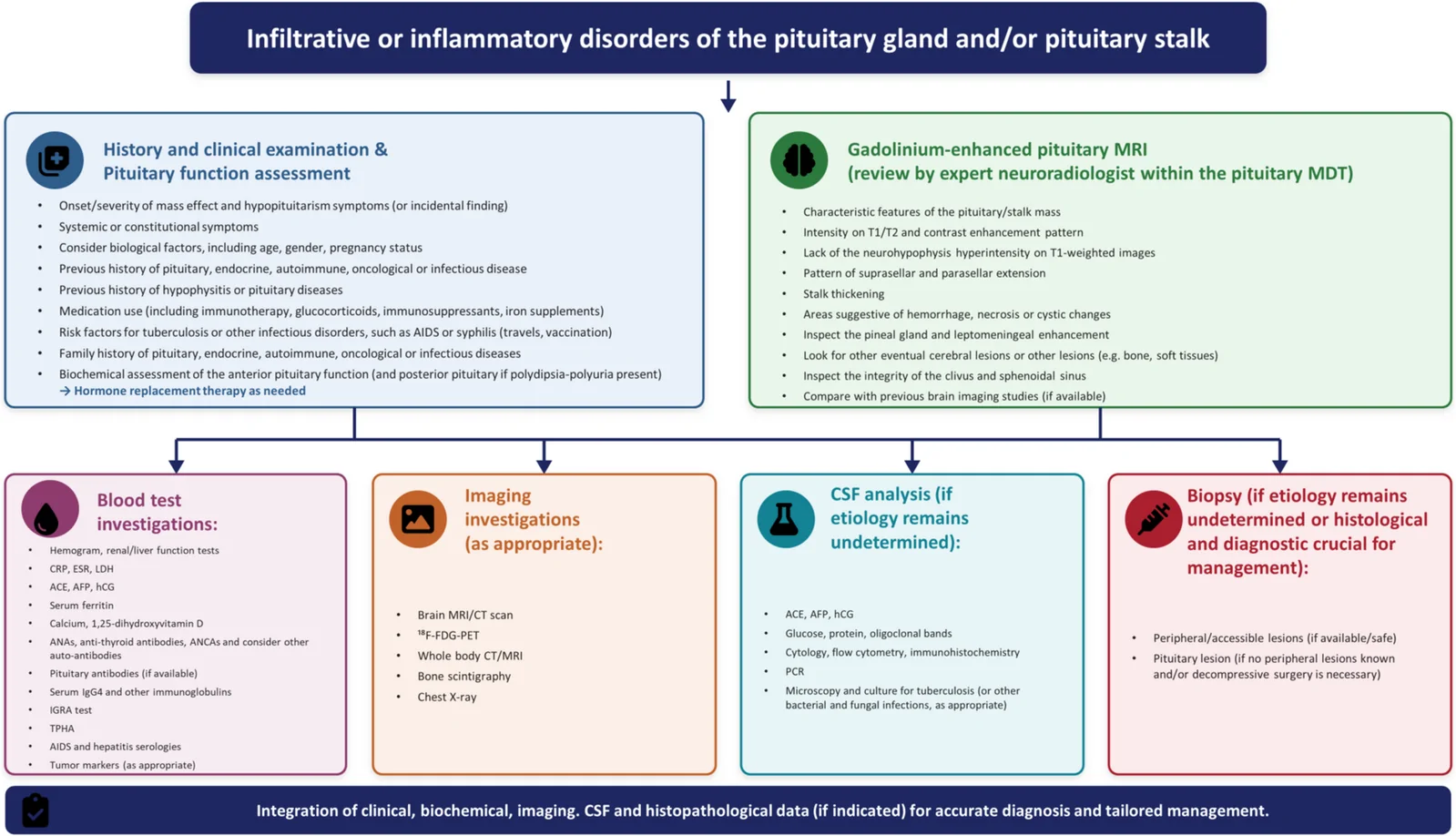
Diagnostic algorithm for a patient with a suspected infiltrative or inflammatory disorder of the pituitary gland. Abbreviations: ACE, angiotensin-converting enzyme; AFP, alpha-fetoprotein; AIDS, acquired immunodeficiency syndrome; ANAs, antinuclear antibody; ANCA, anti-neutrophil cytoplasmic antibody; CRP, C-reactive protein; CSF, cerebrospinal fluid; CT, computed tomography; ds-DNA, double-stranded DNA; ESR, erythrocyte sedimentation rate; FDG-PET, fluorodeoxyglucose positron emission tomography; hCG, human chorionic gonadotropin; IgG4, immunoglobulin G4; IGRA, interferon-gamma release assays; LDH, lactic acid dehydrogenase; MDT, multidisciplinary team; MRI, magnetic resonance imaging; SSA, Sjögren’s-syndrome-related antigen A; SSB, Sjögren’s-syndrome-related antigen B; TPHA, Treponema pallidum hemagglutination assay

The diagnostic work-up should follow a structured, stepwise algorithm (Fig. 6). The initial evaluation combines a detailed history and clinical examination. Gadolinium-enhanced pituitary MRI, reviewed by an expert neuroradiologist within a pituitary multidisciplinary team, should characterize the pattern of pituitary/stalk enlargement and thickening, T1/T2 signal and enhancement pattern, loss of the posterior pituitary bright spot, suprasellar/parasellar extension, hemorrhagic, necrotic, or cystic change, pineal or leptomeningeal involvement, and the integrity of the clivus and sphenoid sinus, compared against prior imaging when available. Targeted blood tests, should be guided by clinical context rather than performed indiscriminately. When the etiology remains undetermined, cerebrospinal fluid analysis (ACE, AFP, hCG, glucose, protein, oligoclonal bands, cytology, flow cytometry, immunohistochemistry, PCR, and microscopy/culture for tuberculosis or other organisms) and systemic imaging (chest X-ray, whole-body CT/MRI, bone scintigraphy, and ¹⁸F-FDG-PET) help identify a more accessible biopsy site and avoid unnecessary pituitary surgery [104]. Histopathological confirmation, of a peripheral or accessible lesion when available and safe, or of the pituitary lesion itself when no peripheral site is identified or decompressive surgery is otherwise required, remains the diagnostic gold standard and should be pursued whenever the diagnosis stays uncertain after non-invasive evaluation, when malignancy cannot be excluded, or before committing a patient to long-term immunosuppression.

Neuroimaging findings, while rarely pathognomonic and overlap considerably, should always be interpreted alongside the hormonal, systemic, and laboratory data.

Management follows two parallel tracks. First, hormone replacement therapy should be initiated promptly for any confirmed deficiency; corticotroph deficiency in particular requires urgent treatment given its life-threatening potential, regardless of the underlying etiology. Second, disease-specific therapy should be tailored to the confirmed diagnosis: glucocorticoids [105] and, when needed, steroid-sparing immunosuppressants or rituximab for autoimmune and IgG4-related hypophysitis; disease-modifying agents (glucocorticoids, cyclophosphamide, or rituximab) for granulomatous and vasculitic disorders; targeted antimicrobial or antituberculous therapy for infectious causes; and BRAF/MEK inhibitors, chemotherapy, or radiotherapy for histiocytic and neoplastic disorders. Surgery should be reserved for diagnostic uncertainty, significant mass effect with visual or neurological compromise, or failure of medical therapy, since pituitary function rarely recovers fully regardless of the treatment modality used. Because pituitary hormone deficits are frequently irreversible and recurrence is common across most of these disorders, long-term endocrine follow-up with serial hormonal testing and periodic MRI is warranted in virtually all patients. Follow-up should not be limited to hormonal surveillance alone: several of these conditions follow a relapsing-remitting course, and patients remain at risk of disease flares or reactivation, at the pituitary level or at extrapituitary sites, even after apparent radiological and hormonal stabilization. Long-term monitoring should therefore also incorporate periodic clinical reassessment and, when applicable, disease-specific markers (e.g., serum IgG4, ANCA titers, ACE levels) and systemic imaging, allowing early detection of relapse before it causes further hypothalamic-pituitary or extrapituitary damage. A multidisciplinary approach, involving endocrinology, neurosurgery, neuroradiology, pathology, and the relevant medical or oncological subspecialty, is therefore crucial for timely recognition, accurate diagnosis, and appropriate, disease-specific management of infiltrative and inflammatory hypothalamic-pituitary disorders.

## Data Availability

No datasets were generated or analysed during the current study.
